# Expression of a Functional Recombinant Human Basic Fibroblast Growth Factor from Transgenic Rice Seeds

**DOI:** 10.3390/ijms14023556

**Published:** 2013-02-07

**Authors:** Na An, Jiquan Ou, Daiming Jiang, Liping Zhang, Jingru Liu, Kai Fu, Ying Dai, Daichang Yang

**Affiliations:** 1 Engineering Research Center for Plant Biotechnology and Germplasm Utilization, Ministry of Education, State Key Laboratory of Hybrid Rice, College of Life Sciences, Wuhan University, Wuhan 430072, China; E-Mails: anna_8596@163.com (N.A.); zlp79717@yahoo.com.cn (L.Z.); alexfk1226@yahoo.cn (K.F.); 2 Wuhan Institute of Biotechnology, Biolake, Wuhan 430075, China; E-Mails: jqou@oryzogen.com (J.O.); jdmay1986@126.com (D.J.); jingruliu@oryzogen.com (J.L.); dying@oryzogen.com (Y.D.); 3 Healthgen Biotechnology Ltd. Co., Biolake, Wuhan 430075, China

**Keywords:** recombinant human bFGF, rice endosperm, protein expression and processing, cell proliferation and wound healing

## Abstract

Basic fibroblast growth factor (FGF-2) is an important member of the *FGF* gene family. It is widely used in clinical applications for scald and wound healing in order to stimulate cell proliferation. Further it is applied for inhibiting stem cell differentiation in cultures. Due to a shortage of plasma and low expression levels of recombinant rbFGF in conventional gene expression systems, we explored the production of recombinant rbFGF in rice grains (*Oryza sativa* bFGF, OsrbFGF). An expression level of up to 185.66 mg/kg in brown rice was obtained. A simple purification protocol was established with final recovery of 4.49% and resulting in a yield of OsrbFGF reaching up to 8.33 mg/kg OsrbFGF. The functional assay of OsrbFGF indicated that the stimulating cell proliferation activity on NIH/3T3 was the same as with commercialized rbFGF. Wound healing *in vivo* of OsrbFGF is equivalent to commercialized rbFGF. Our results indicate that rice endosperm is capable of expressing small molecular mass proteins, such as bFGF. This again demonstrates that rice endosperm is a promising system to express various biopharmaceutical proteins.

## 1. Introduction

Fibroblast growth factors (FGFs) are a family of proteins that is structurally related to heparin-binding polypeptides with similar biological activities. To date, 23 different FGFs have been isolated [1]. A prototype of FGFs: *i.e.*, bFGF (basic fibroblast growth factor, bFGF or FGF-2) was reported in the bovine brain in 1978 [2], and the first authentic purification of FGF-2 was carried out in 1984 [3]. It is a single-strand protein with a molecular weight of ~17 kDa and a pI of 9.6. It can stimulate the proliferation of NIH/3T3 cells and inhibit the differentiation of stem cells, and may play an important role in curing asthma because of its capacity for proliferation, migration and changing the contractile phenotypes of human airway smooth muscle cells *in vitro* [4]. Subsequently, bFGF has been found to stimulate the growth of mouse mammary epithelial cells [5]. In clinical applications it is widely used for acceleration of scald and wounding healing. Treatment of wounds showing redness, skin elevation and scald sealing with bFGF is superior to wounds caused by surgery, and there is also less skin hardness [6]. Therefore, bFGF is also used for effective tissue repair and wound healing of cardiovascular and neurodegenerative diseases.

bFGF was originally isolated from bovine brain and pituitary. Limited sources of bFGF make it difficult to meet the demand for the large amounts of bFGF required for both *in vivo* and *in vitro* applications. Over the past few decades, extensive efforts have been made to express recombinant human bFGF in *E. coli*, *Pichia pastoris*, soybean seeds, silkworm (*Bombyx mori* L.) and *Bacillus subtilis* [7–11]. Low yields, complicated processing and insolubility have been encountered when meeting the demand of the markets, especially, with an increasing number of applications for cell therapy and translational medicine. Recently, rice endosperm has been reported to express various recombinant pharmaceutical proteins, including human lactoferrin [12], human lysozyme [13], rhIGF-1 fusion, human granulocyte-macrophage colony stimulating factor [14] and human serum albumin [15]. These results showed that it is cost effective, safe, easy to scale-up and simpler to process than animal and other plant cell expression systems. Therefore, rice endosperm is considered to be a favorable expression platform for large-scale production of pharmaceutical proteins. The advantages are attractive for both research and industrial applications [16].

Here, we successfully expressed recombinant bFGF in rice (Oryza sativa) endosperm (OsrbFGF) with expression levels reaching 99.11–185.66 mg/kg of brown rice. A resulting yield of up to 8.33 mg/kg of brown rice, a purity >95% and a 4.49% recovery were obtained. A simple purification protocol from rice seed was established. Further investigations indicate that OsrbFGF provides the same stimulation of NIH/3T3 cell proliferation. The purified OsrbFGF has the same efficacy of wound healing *in vivo* as other tested products.

## 2. Results

### 2.1. Generation and Selection of the Transgenic Lines for High Expression of OsrbFGF in Rice Seeds

**Figure 1:**
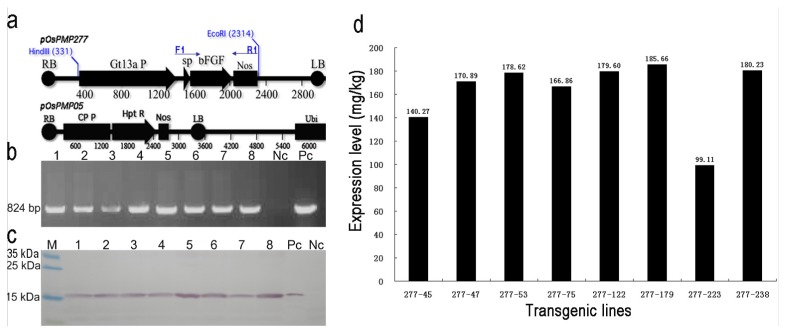
Generation and screening of the transgenic plants highly expressing recombinant basic fibroblast growth factor (bFGF) in rice (Oryza sativa) endosperm (OsrbFGF): (**a**) Maps of plasmids of *pOsPMP277* and *pOsPMP05*; (**b**) Agarose gel for screening positive transgenic lines by polymerase chain reaction (PCR) amplification with primers F1 and R1 and (**c**) identification of the transgenic lines from T1 seed expressing OsrbFGF by Western blotting; (**d**) Quantitation of expression levels of the individual T1 transgenic seeds. Lane M, marker; Lane 1, 277–45; Lane 2, 277–47; Lane 3, 277–53; Lane 4, 277–75; Lane 5, 277–122; Lane 6, 277–179; Lane 7, 277–223; Lane 8, 277–238; Pc, plasmid *pOsPMP277* (**b**) and 350 ng of recombinant bFGF from *E. coli* (**c**); Nc, wild type TP309 DNA (**b**) and crude protein extracts from the seed of wild type TP309 (**c**).

An endosperm-specific promoter from a glutelin gene *Gt13a* and its signal peptide were used in this study [14,17]. A human mature basic fibroblast growth factor (bFGF) gene was optimized with a rice codon bias. A total of 58 independent transformants was obtained using a two-strain *Agrobacterium*-mediated transformation [18] (Figure 1a). The positive transgenic plants were identified by polymerase chain reaction (PCR) analysis with specific primers (Figure 1b). Western blotting indicated that 35 (63.34%) transformants accumulated OsrbFGF in the rice endosperm (Figure 1c). The expression level of total OsrbFGF ranged from 99.11 to 185.66 mg/kg of brown rice from eight lines with high fertility (Figure 1d). Transgenic lines, 277–122 and 277–238, were the highest expressing lines and were used for further study.

### 2.2. Genetic Analysis of the Transgenic Lines and Characterization of OsrbFGF

**Figure 2:**
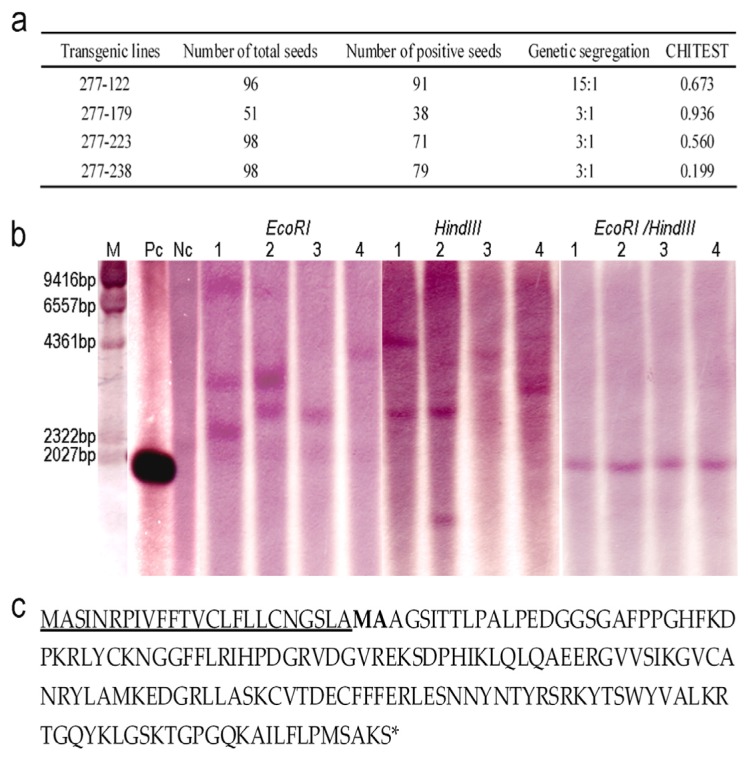
Genetic analyses of the transformants by transgene segregation and Southern blotting: (**a**) Genetic segregation of T_1_ seeds from transgenic lines; (**b**) Southern blot analysis of four transgenic lines and TP309. Genomic DNA was digested with *Eco*RI or *Hind*III and *Eco*RI*/Hind*III, and plasmid *pOsPMP277* and wild type TP309 genomic DNA were digested with *Eco*RI*/Hind*III. Lane 1, 277–122; Lane 2, 277–179; Lane 3, 277–223; Lane 4, 277–238; M, marker (Lambda DNA digested by *Hind*III); Pc, positive control (*pOsPMP277* DNA with length of 1983bp); Nc, negative control (Wild-type TP309 genomic DNA); (**c**) Amino acid sequences of the signal peptide and OsrbFGF. The signal peptide is underlined and two missed amino acids (Met and Ala) are in bold.

The segregation for transgene expression was determined in the T1 seeds of the transgenic lines 277–238, 277–223, 277–122 and 277–179, genetic segregation in T1 seeds based on the expression: non-expression was analyzed. The results showed that 277–238, 277–223 and 277–179 showed a single locus and 277–122 two loci (Figure 2a). Southern blotting was carried out for further characterization of copy numbers in these transgenic lines. The results indicated that one fragment from transgenic lines 277–238 and 277–223 was detected by digestion of either *Eco*RI or *Hind*III, which is consistent with the results from genetic segregation. Digestion with *Eco*RI and *Hind*III of 277–122 yielded three fragments and two fragments, respectively. The results of genetic segregation and Southern blots of 277–122 indicated that it contained two loci. Although the transgenic line 277–179 showed two loci when digested with *Eco*RI or *Hind*III, the fragment obtained with *Hind*III was of smaller size to the entire expression cassette obtained with double digestion with *Hind*III/*Eco*RI. This suggested that it was a truncated locus. This further explained why 277–179 yielded two loci with Southern blotting, while genetic segregation of expression indicated a single locus (Figure 2 a,b). To further determine whether rice endosperm cells can properly cleave the signal peptide at the *N*-terminus of OsrbFGF, *N*-terminal analysis was carried out and revealed that two amino acids, Met and Ala, were missing at the *N*-terminus of OsrbFGF (Figure 2c).

### 2.3. Purification of OsrbFGF from Transgenic Rice Seeds

**Figure 3:**
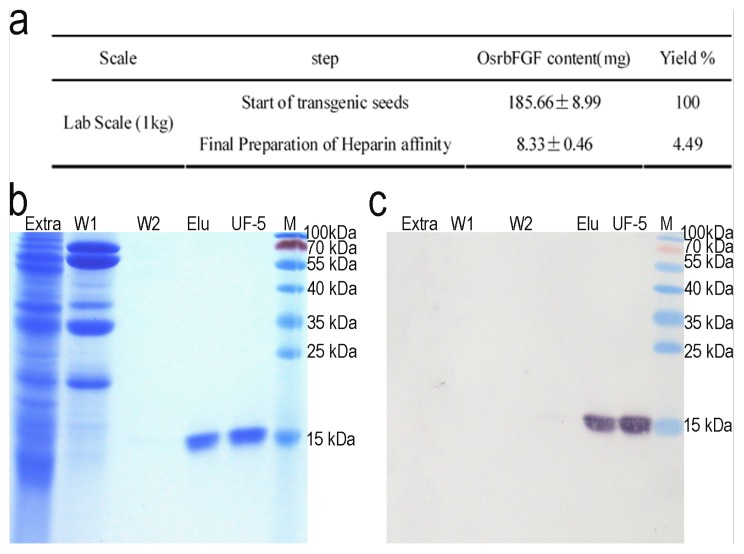
Purification of OsrbFGF from the seeds derived from transgenic line 277–179. (**a**) The recovery rate of OsrbFGF for purification step; (**b**) sodium dodecyl sulfate polyacrylamide gel electrophoresis (SDS-PAGE) with Coomassie blue G250 staining for each processing step; (**c**) Western blotting analysis of each processing step. Extra: the crude extraction; W1: wash 1; W2: wash 2; Elu: Elutant; UF-5: ultrafiltration in size of 5 kDa; M: molecular mass marker.

After the highly expressing OsrbFGF transgenic lines were identified, we developed a purification protocol for large scale processing of OsrbFGF that could satisfy the demand of the markets. We firstly tried to separate the impurities using different ion exchange resins from rice seeds; however, all efforts failed (data not shown). Then we tried direct application of a heparin affinity column and washing with two different buffers that removed most of the impurities from the affinity column. A high purity OsrbFGF with a single band of 17 kDa was obtained (Figure 3). Endotoxin contamination was removed with one step of ultrafiltration using 5 kDa or 100 kDa filters. The resulting purity of OsrbFGF reached up to >95% and <1 EU/μg, respectively. The final yield of OsrbFGF was 8.33 mg/kg of brown rice with a recovery of 4.49%.

### 2.4. OsrbFGF Can Effectively Stimulate NIH/3T3 Cell Proliferation

**Figure 4:**
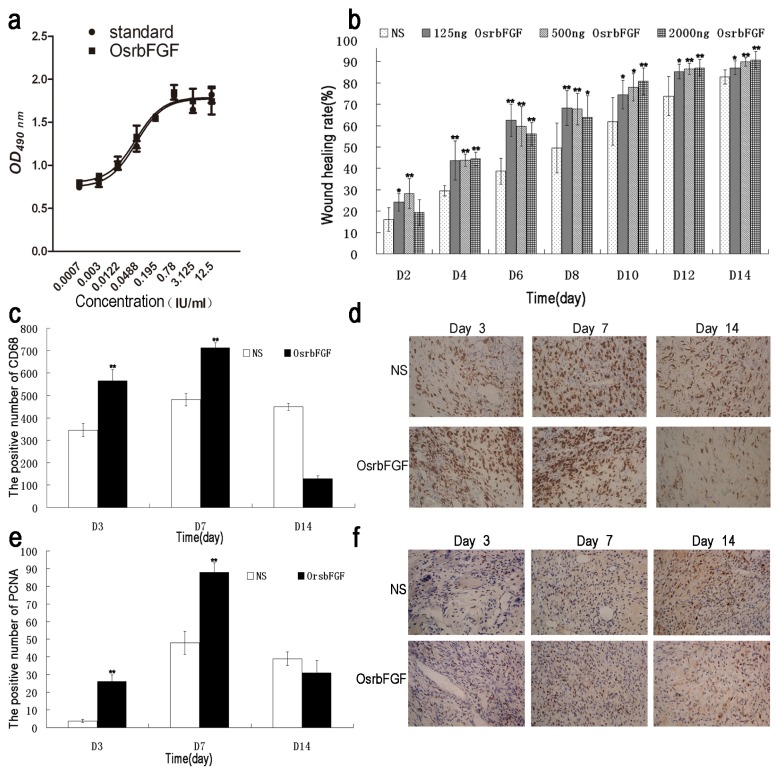
Biological activity assay of OsrbFGF *in vitro* and *in vivo*. (**a**) The cell proliferation assay of OsrbFGF with NIH/3T3 cells; (**b**) Wound healing rate on Day 2, Day 4, Day 6, Day 8, Day 10, Day 12 and Day 14. The diagrams of the positive cells of CD68 (**c**) and proliferation of cell nuclear antigen (PCNA) (**e**) on Day 3, Day 7 and Day 14 (*t*-test *p < 0.05, **p < 0.01). The microimages of immune autoradiography of (**d**) CD68 and (**f**) PCNA (x40). For the immunohistochemistry assay, a dosage of 500 ng OsrbFGF was chosen and the positive cells with CD68 and PCNA were counted. NS means normal saline.

The NIH/3T3 cell line is generally used for evaluation of the biological activity of FGFs [19]. To evaluate the biological activity of OsrbFGF, stimulation of NIH/3T3 cells proliferation was tested. As shown in Figure 4a, OsrbFGF concentrations from 0.0007 to 12.5 IU/mL showed a dose-dependent cell proliferative effect on NIH/3T3 cells, reaching 1.04 × 10^6^ IU/mg. The result suggests that OsrbFGF has the same stimulation of cell proliferation as commercialized rbFGF.

### 2.5. OsrbFGF Has Equivalent Efficacy in Wound Healing

bFGF is used for would healing therapy [20]. To evaluate its biological activities *in vivo*, OsrbFGF was applied to a mouse cut model for wound healing. Three dosages of OsrbFGF; *i.e.*, 125 ng/treatment, 500 ng/treatment and 2000 ng/treatments were tested. Wound healing rates were observed on Day 0, Day 2, Day 4, Day 6, Day 8, Day 10, Day 12 and Day 14 after wound treatments. The results indicated that wound healing rates were dose-dependent. The efficacy of stimulating wound healing at a low-dose was more significant than that of 500 ng and 2000 ng within eight days after treatment (Figure 4b). In general, the recovery of wounded skin could promote epithelialization, increase expression of the CD68 and proliferation of cell nuclear antigen (PCNA) [21]. We therefore monitored the expression of PCNA and CD68 on the wound area on Day 3, Day 7 and Day 14 treatments from a dosage of 500 ng OsrbFGF. The results showed that CD68 and PCNA in the OsrbFGF treatments were increased significantly compared to that of the negative control on Day 3 and Day 7 of post-wound healing and was lower than that of post-wound healing in the control group on Day 14 (Figure 4 d,f). The results indicated that OsrbFGF could accelerate wound healing by stimulating cell proliferation and improving the expression of CD68 and PCNA in the early stage of treatment. It then decreased the expression to prevent the generation of scars and cell proliferation when the wound closed at the late stage of the treatment.

## 3. Discussion

In general, small growth factors are usually difficult to express in current expression systems because they have a small molecular mass. Our study demonstrated that rice endosperm cells as bioreactors successfully expressed functional OsrbFGF and that the expression levels of total OsrbFGF reached up to 185.66 mg/kg in brown rice, of which 17.74 mg/kg was soluble OsrbFGF accounting for 9.55% of total OsrbFGF (data not shown). The yield was two- to four-fold higher than that in *E. coli*, *Bacillus subtilis*, insect cell and yeast (*Saccharomyces cerevisiae*) systems [7,8,22,23], but lower than that in *Pichia pastoris* systems [11]. Although only 4.49% of the total OsrbFGF was recovered due to the insolubility of OsrbFGF, the resulting yield still reached 8.33 mg/kg in brown rice, which was significantly higher than the required threshold for commercialization. Furthermore, we successfully developed a simple purification protocol to isolate OsrbFGF from rice grains, demonstrating that rice grains have the advantages over other systems for recombinant protein processing. In addition, two amino acids at the *N*-terminus of OsrbFGF were deleted but the protein retained the same biological functions both *in vitro* and *in vivo*. This suggested that the two amino acids at the *N*-terminus were not essential for its function.

In previous reports, rice endosperm cells have been demonstrated to be capable of expressing various pharmaceutical proteins [12–15,17]. Our results showed that rice endosperm cells had the capability of expressing proteins with special features of proteins, such as small molecular mass and hydrophobic proteins. Most of the OsrbFGF was soluble when detergent and reduced reagents were used, which implied that OsrbFGF could be misfolded or aggregated. To solve this problem, one approach could be via co-expression of molecular chaperons to promote help with exogenous protein folding and to prevent aggregation. Most transgenic endosperms with high recombinant protein expression developed endoplasmatic reticulum (ER) stress [24]. Another approach to increase the solubility of recombinant proteins in rice endosperm cells could be to decrease the expression of endogenous storage proteins to alleviate the ER stress.

## 4. Experimental Procedures

### 4.1. Plasmid Construction and Rice Transformation

*pOsPMP02* was used to construct an endosperm-specific expression cassette [14]. The coding sequence of the human *bFGF* gene (Genbank accession No. NM 002006) was synthesized by Heron Blue Biotechnology Inc. (Bothell, WA, USA) using rice-preferred genetic codons. The synthesized gene was cleaved by *Sch*I and *Xho*I and then cloned into *pOsPMP02* to produce *pOsPMP276*. A binary vector for *Agrobacterium*-mediated transformation was produced by digesting *pOsPMP276* with *Hind*III and *Eco*RI and an entire expression cassette with a 1983-bp fragment containing the *Gt13a* promoter and the signal peptide, codon optimized *bFGF* gene and *Nos* terminator was inserted into a binary vector *JH2600* [25]. The resulting binary plasmid was designated as *pOsPMP277*. The binary plasmid was transferred into the *Agrobacterium tumefaciens* strain *EHA105* harboring plasmid *pOsPMP05* containing a selective marker gene [14], and *pOsPMP277* were co-transformed into the calli regenerated from a rice variety TP309 by the *Agrobacterium*-mediated transformation as described previously [26].

### 4.2. Screening for High Expressing OsrbFGF Transgenic Lines and Determination of the Expression Level

To screen the positive transgenic lines, a forward primer F1 (5′-GAGGGTGTGGAGGCTCTTGT-3′) from the *Gt13a* signal peptide and a reverse primer R1 (5′-GCCAGTGAATTCCCGATCTAGTAAC-3′) from the *Nos* terminator were used for PCR amplification.

To obtain the expression levels of transgenic lines, Western blot was used. Briefly, 100 mg of T1 seeds from a PCR positive transgenic plant were ground with 1 mL of extraction buffer (66 mM Tris-HCl, pH 6.8, 2% SDS, 1 mM dithiothreitol (DTT)), and then centrifuged at 10,620× *g* for 10 min. Fifteen micrograms of the crude protein extracts and 350 ng of recombinant bFGF derived from *E. coli* were separated by a 15% SDS-PAGE gel and then transferred to a nitrocellulose membrane. A rabbit polyclonal to bFGF (Abcam, Cambridge, UK) in a dilution of 1:2000 and alkaline phosphatase goat anti-rabbit IgG (ZSGB-BIO, Beijing, China) in a dilution of 1:10000 were used. Detection of target protein was carried out using the substrates p-Nitro-Blue Tetrazolium Chloride and 5-Bromo-4-Chloro-3-Indolyl Phosphate (BIOSHARP, Hefei, China). Immunoblotting procedures were performed as described previously [17]. The expression level was determined from Western blotting by the Image-pro Plus software. Briefly, four concentrations of 50 ng, 100 ng, 200 ng and 400 ng of rbFGF derived from *E. coli* were used for quantitation of OsrbFGF level. The density of each known concentrations of rbFGF in Western blot was obtained by Image-pro Plus software. The expression level of each transgenic line was calculated based on the equation *y* = 2.0018* − 118.05 (*R*^2^ = 0.98722).

### 4.3. Southern Blotting

About 200 milligrams of young leaf were ground with liquid nitrogen, and genomic DNA extraction was carried out using a TIANGEN DNA Quick Plant System (TIANGEN Biotech, Beijing, China). Genomic DNA was digested with *Eco*RI, *Hind*III, or *Eco*RI/*Hind*III (New England Biolabs, Ipswich, MA, USA), respectively, at 37 °C overnight and then was separated by 0.8% agarose gel. After transfer to a MILLIPORE NY + membrane, hybridization was carried out following the instructions of Roche DIG High Prime DNA Labeling and Detection Starter Kit I. A probe with an 824 bp fragment derived from the *bFGF* encoding region was prepared by amplifying with the primers F1 and R1.

### 4.4. OsrbFGF Purification

The transgenic rice seeds were ground and extracted (1:5, *w*/*v*) with the extraction buffer (50 mM phosphate buffer, pH 7.5, 1 mM EDTA, 1 mM reduced L-Glutathione, 250 mM NaCl) for 1 h at room temperature. Then, the crude extract was clarified by the following 3 μm and 0.22 μm filtration through a positive pressure filter, respectively. Subsequently, the filtrate was applied onto the heparin 6 fast flow (GE Healthcare, www.gelifesciences.com) column equilibrated with 50 mM phosphate buffer (pH 7.5) containing 250 mM NaCl and 1 mM reduced l-glutathione. Two wash steps were used to remove the impurity proteins and increase the OsrbFGF recovery. Wash buffer 1 (50 mM phosphate buffer (pH 7.5), 600 mM NaCl, 1 mM reduced l-glutathione) and Wash buffer 2 (50 mM phosphate buffer (pH 7.5), 900 mM NaCl, 1 mM reduced l-glutathione) were used to remove impurities. After washing, the OsrbFGF was eluted by elution buffer (10 mM Tris-HCl (pH 7.5), 1.7 mM NaCl, 1 mM reduced l-glutathione). Before lyophilization, the OsrbFGF elution was concentrated and partially desalted using a 5 kD ultrafiltration filter with Pellicon XL (Millipore, Billerica, MA, USA), and then endotoxins were removed by a 100 kD ultrafiltration filter with a Viva Flow 50 (Sartorius, Goettingen, Germany). Finally, OsrbFGF was lyophilized with the protection of OsrHSA (1:50, *w*/*w*) [15]. The final protein concentration was determined using the Coomassie Plus (Bradford) Assay Kit (Thermo Fisher Scientific Inc., Rockford, IL, USA).

### 4.5. *N*-terminal Sequence Analysis

The purified OsrbFGF was transferred to a polyvinylidene difluoride (PVDF) membrane and then put into a protein sequencer PPSQ-31A (Shimadzu Cooperation, Kyoto, Japan) reactor to run 15 cycles.

### 4.6. MTT Assay

The mitogenic activity of OsrbFGF was assessed through NIH/3T3 cell proliferation using the methylthiazoletetrazolium (MTT) method. NIH/3T3 cells were seeded in flat-bottom, 96-well plates at an initial density of 2 × 10^5^ cells per mL (200 μL per well) and cultured in Dulbecco’s modified eagle minimum essential medium (DMEM) supplemented with 10% FBS for 24 h and then cultured in fresh maintenance DMEM containing 0.75% FBS for 24 h. After the medium was removed, 200 μL maintenance medium containing a four-fold serial dilution of OsrbFGF or the recombinant bFGF standard with the initial concentration of 50 IU/mL was added into the wells and the cells were incubated for 48 h. The number of viable cells was determined by adding 20 μL methylthiazoletetrazolium (MTT 5 mg/mL) to each well. The culture was incubated for 4 h. After removal of the media, 150 μL dimethyl sulfoxide (DMSO) was added to each well. The plate was kept at room temperature for 30 min and was measured at 490 nm in a VersaMax 400 nm (Molecular Devices, Sunnyvale, CA, USA). All the data were analyzed with the GraphPad software. All experiments were repeated three times.

### 4.7. Wound Healing Assay

Female Sprague-Dawley rats (SD) with 200–250 g of body weight were obtained from Shanghai Slac Laboratory Animal Co. Ltd. The SD rats were randomly divided into four groups, and the excisional wound splinting model was generated as described previously [27]. Three dosages of OsrbFGF of 2000 ng/mL (*n* = 15), 500 ng/mL, (*n* = 15), 125 ng/mL, (*n* = 15) and negative control with normal saline (*n* = 15) were designed for testing the healing rates. The wound healing rate = (Area of original wound − Area of actual wound)/Area of original wound × 100.

### 4.8. Immunohistochemistry Assay

For the immunohistochemistry assay, a dosage of 500 ng OsrbFGF was chosen. Fixation, sectioning and immuno-electronic microscopic observations followed the procedure as previously described [28]. A mouse antibody against CD68 or PCNA in a dilution of 1:100 and a goat anti-mouse IgG-HRP in a dilution of 1:200 were used. Finally, each slide was stained with the diaminobenzidine (DAB) substrate. After immunofluorescence staining, CD68 or PCNA positive cells were counted from three fields per section at the wound site between the edges in six successive sections.

## 5. Conclusions

We successfully expressed functional recombinant bFGF (*Oryza sativa* bFGF, OsrbFGF) in rice endosperm cells and established a simple purification protocol with single step processing. The results indicate that the stimulating cell proliferation activity on NIH/3T3 of OsrbFGF and the acceleration of wound healing *in vivo* are comparable to commercialized rbFGF. Our results indicate that rice endosperm is capable of expressing high hydrophobic proteins, such as bFGF. Our data again demonstrates that rice endosperm is a promising system to express various biopharmaceutical proteins.
